# An updated meta-analysis evaluating limb management after total knee arthroplasty—what is the optimal method?

**DOI:** 10.1186/s13018-019-1140-y

**Published:** 2019-04-10

**Authors:** Hai-yang Wang, Guang-shu Yu, Jie-hui Li, Shou-xiong Zhang, Yan-bin Lin

**Affiliations:** Department of Orthopedics, Fuzhou the Second Hospital Affiliated to Xiamen University, 47 Shangteng Road, Fuzhou, 350,007 Fujian People’s Republic of China

**Keywords:** Total knee arthroplasty, Mild flexion, High flexion, Duration of flexion, Blood loss, Range of motion

## Abstract

**Purpose:**

Postoperative knee flexion protocol has been widely recognized as a highly attractive, simple, and cost-effective tactic to improve patient’s outcomes after primary total knee arthroplasty (TKA). However, optimal knee position and duration of knee flexion are still controversial. The purpose of this meta-analysis was to compare the effectiveness of different postoperative knee flexion protocols, as an aid to find out optimal limb management strategy following TKA.

**Methods:**

We conducted a meta-analysis to identify the available and relevant randomized controlled trials (RCTs) with regard to the influence of different postoperative knee positions on clinical outcomes after primary TKA in electronic databases, including PubMed, EMBASE, the Cochrane Library, Web of Science, CNKI, Wanfang Med Online, and VIP, up to May 2018. In this meta-analysis, three major subgroups based on diverse postoperative knee flexion protocols were considered: long-term (≥ 24 h) high flexion (> 30°), short term (< 24 h) high flexion (> 30°), and long-term (≥ 24 h) mild flexion (≤ 30°). The statistical analysis was performed using the Review Manager (RevMan) version 5.3 software.

**Results:**

A total of 16 trials were finally included in this meta-analysis. The result of subgroup analysis indicated that keeping the knee in high flexion (> 30°) postoperatively for a long time (≥ 24 h) significantly reduced total blood loss (*P* < 0.00001), hidden blood loss (*P* < 0.00001), and transfusion requirements (*P* = 0.003) and led to a significant improvement in range of motion (ROM) at 1 week after operation (*P* < 0.00001); keeping the knee in high flexion (> 30°) postoperatively for a short time (< 24 h) significantly reduced total blood loss (*P* = 0.006) and hidden blood loss (*P* < 0.00001) but not significantly improved ROM at 1 week after operation (*P* = 0.34) and reduced transfusion requirements (*P* = 0.62); and keeping the knee in mild flexion (≤ 30°) postoperatively for a long time (≥ 24 h) significantly reduced total blood loss (*P* = 0.02) and transfusion requirements (*P* = 0.02) and improved ROM at 1 week after operation (*P* < 0.00001) but not significantly reduced hidden blood loss (*P* = 0.11). Furthermore, there was no significant difference with respect to the rates of wound-related infection and DVT between the three knee flexion subgroups.

**Conclusions:**

This meta-analysis showed that the long-term (≥ 24 h) high flexion (> 30°) protocol could be an optimal limb management to reduce blood loss and blood transfusion requirements and facilitate early postoperative rehabilitation exercises in patients after primary TKA without increasing in complication rate.

## Introduction

Total knee arthroplasty (TKA) is an effective surgical treatment method for patients with moderate to severe arthritis who has not responded to medical management [1]. However, surgical interventions can be associated with substantial blood loss, which may result in anemia and require postoperative blood transfusion. Previous studies reported blood loss related to TKA can exceed 1000 mL [2], approximately 20% of the body’s total blood volume [3], and the rate of blood transfusion following TKA can be as high as 40% [4, 5]. Allogenic blood transfusion could not only increase the likelihood of transmission of disease, immunological reactions, transfusion-associated circulatory overload, and even death [6, 7] but could also prolong the duration of hospital stay and increase medical costs [8, 9]. Most importantly, blood loss also affects the recovery of knee range of motion (ROM), but the restoration of a satisfactory ROM is crucial for an optimal result, and slight improvements in maximum flexion can have profound effects on functional capability [10].

Thus, various prevention agents and techniques have been proposed to reduce postoperative blood loss and subsequent transfusion requirements, such as various drainage protocols, tourniquet use, pharmacological methods (fibrin spray, adrenaline, and tranexamic acid), postoperative cryotherapy and knee positioning, and invasive and computer-aided surgical techniques [11–18]. Among these, keeping the knee in flexion postoperatively has been identified as a highly simple and cost-effective way to reduce blood loss and transfusion requirements and increase ROM after primary TKA [19–26]. Nevertheless, there are opposite opinions about the availability and safety of postoperative knee positioning on outcomes after TKA [27–29].

Two previous meta-analyses have compared the impact of flexion versus extension of knee position on outcomes after TKA and demonstrated that positioning the knee in flexion was associated with significantly lesser total blood loss, lesser hidden blood loss, decreased need for blood transfusion, and better ROM in the early postoperative period [7, 30]. Thus far, there is no clear consensus upon whether it is accurate knee position and duration of knee flexion which conduces most to the benefits seen with postoperative knee flexion strategies. A previous systematic review has shown that keeping the knee in flexion postoperatively for 48–72 h appears to be effective in reducing blood loss and increasing ROM following TKA, and the application of shorter (6–24 h) post-operative knee flexion strategies reveals no benefit [31]. However, recently, three high-quality randomized controlled trials (RCTs) reported knee flexion for 6–24 h could significantly reduce blood loss and the need for blood transfusion compared with knee extension following TKA [20, 25, 26]. A recent updated meta-analysis of RCTs has demonstrated that compared with knee high flexion (≥ 60°), mild flexion (<60°) positioning is significantly beneficial with hidden blood loss after TKA and high flexion (≥60°) positioning is superior to mild flexion (< 60°) positioning in reducing blood transfusion requirements and improving ROM following TKA [32], but the study did not perform subgroup analysis based on the time (6–72 h) of knee flexion, which may lower the accuracy of conclusions. Although the results of new RCTs by De Fine et al [33] suggested that no significant differences related to blood loss reduction, blood transfusion diminution, and ROM improvement after TKA were found between the high flexion (70°) and mild flexion (30°) group, this study lacked a control group of knee extension and sufficient sample size. In 2018, one new RCT has been published, with seemingly mixed results [26]. In addition, six RCTs [34–39] written in Chinese were not part of previous reviews, which would have brought about statistical bias and publication bias. Furthermore, because of the limited amount of included studies, publication bias failed to be assessed exactly in previous reviews. Hence, we believe those reviews need to be renewed again.

The purpose of the current meta-analysis was to compare the effectiveness and safety of three different knee flexion protocols in patients following primary TKA: long-term (≥ 24 h) high flexion (>30°), short-term (< 24 h) high flexion (> 30°), and long-term (≥24 h) mild flexion (≤ 30°), with regard to total blood loss, hidden blood loss, blood transfusion requirement, ROM at 1 week after operation, deep vein thrombosis (DVT), and wound-related infection, and to eventually find out optimal limb management strategy following TKA.

## Methods

### Search strategies

This study was performed in accordance with the 2009 PRISMA (Preferred Reporting Items for Systematic Reviews and Meta-Analysis) guidelines [40]. A comprehensive literature search was implemented in PubMed, EMBASE, the Cochrane Library, Web of Science, CNKI, Wanfang Med Online, and VIP for RCTs (from the earliest available records to May 1, 2018), using the keywords and their combinations: *total knee arthroplasty*, *TKA*, *total knee replacement*, *TKR*, *knee position*, *leg position*, *limb position*, *extension*, *flexion*. All studies published in English and Chinese were considered for inclusion. Subsequently, a manual search of references of those studies also was conducted by authors for any possibly relevant studies.

### Inclusive and exclusive criteria

Included studies were considered eligible by two authors if they met the following inclusion criteria: (1) RCTs; (2) comparison of knee positioning in flexion versus extension after primary unilateral TKA; (3) trials providing data related to outcomes including total blood loss, hidden blood loss, blood transfusion requirement, ROM at 1 week after operation, DVT, and wound-related infection. Exclusion criteria included review articles, non-randomized trials, quasi-randomized trials, articles involving bilateral TKA and revision knee arthroplasty, and articles with insufficient outcome data. In cases of discrepancy, a consensus was reached through discussion among authors.

### Primary and secondary outcomes

The primary outcomes included total blood loss, hidden blood loss, and transfusion requirement. Secondary outcomes included ROM at 1 week after operation, DVT, and wound-related infection.

### Data extraction

Two authors independently extracted relevant data from each eligible study using a standard data extraction worksheet. Discrepancies in opinion between authors were resolved by discussion and a third author was consulted if necessary. The data extracted included the first author, year of publication, country of origin, participant characteristics, methodological characteristics, type of intervention, surgical procedures, and measured outcomes. If the trials had multiple comparisons, we extracted only the information and data of interest reported in the original trials. We also attempted to contact the corresponding authors of primary studies to make sure the information was integrated and request missing data.

### Quality assessment and risk of bias

The methodological quality of the included RCTs was evaluated independently and carefully by two authors based on the Cochrane Handbook for Systematic Reviews of Interventions, version 5.1.0 (http://handbook.cochrane.org/). The following eight-item scales were assessed: random sequence generation (selection bias), allocation concealment (selection bias), blinding of the participants and personnel (performance bias), blinding of outcome assessments (detection bias), selective reporting (reporting bias), incomplete outcome data (attrition bias), and other biases. Each of the items needed to be measured as “Yes” (low risk of bias), “No” (high risk of bias), or “Unclear” (unclear risk of bias). The risk of bias summary and risk of bias graph were obtained using Review Manager (RevMan), version 5.3 (The Nordic Cochrane Centre, The Cochrane Collaboration, 2009, Copenhagen, Denmark). Disagreements were also settled down by discussion between the two authors.

### Statistical analysis

The statistical analysis was performed with the help of Review Manager (RevMan), version 5.3 (The Nordic Cochrane Centre, The Cochrane Collaboration, 2009, Copenhagen, Denmark), and *P* value <0.05 was regarded as statistically significant. For continuous variables, such as total blood loss, hidden blood loss, and range of motion, mean difference (MD) and 95% confidence interval (*CI*) were calculated. Dichotomous variables such as transfusion requirement, DVT, and wound-related infection were evaluated using risk difference (RD) with a 95% confidence interval (*CI*). Statistical heterogeneity was primary assessed using the *I*^2^ value and chi-square test. If the *I*^2^ < 50% or *P* > 0.05, the heterogeneity might be unconsidered and a fixed effects model was adopted in order to evaluate the outcomes. If *I*^2^ was between 50% and 100%, or if *P* < 0.05, it may show substantial heterogeneity. We used a random effects model to assess these outcomes. To precisely evaluate the influence of different postoperative degree and duration of knee flexion on patient’s outcomes after TKA, we defined short term as flexing < 24 h, long term as flexing ≥ 24 h, high flexion as > 30°, and mild flexion as ≤ 30° [21, 24] and created three major subgroups: long-term high flexion groups, short-term high flexion groups, and long-term mild flexion groups, respectively. In addition, we investigated publication bias by funnel plots when the number of trials reporting the primary outcomes was ten or more [41].

## Results

### Study selection and characteristics of selected studies

**Fig. 1:**
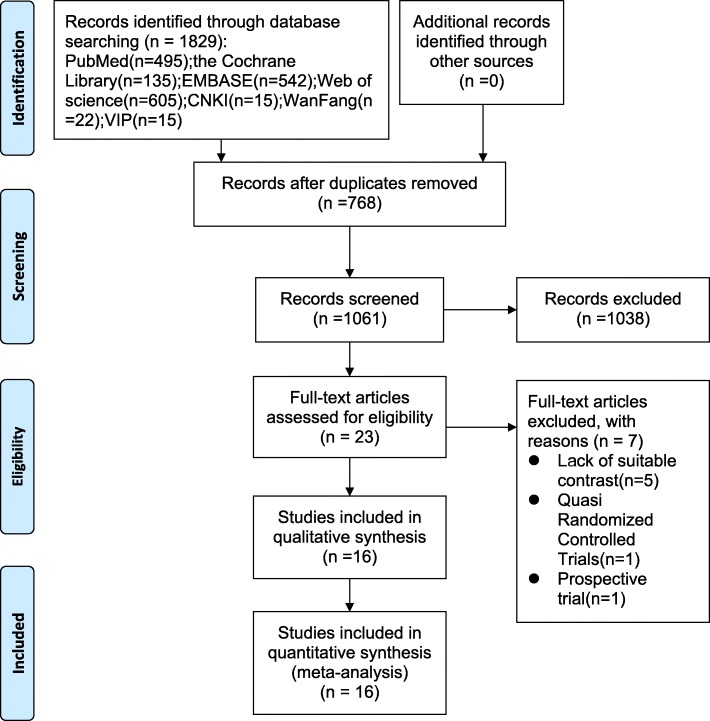
The PRISMA flow diagram detailing our literature search

A total of 1829 relevant studies were initially identified from electronic journals databases. After removal of duplicates, 1061 studies were available for assessing titles and abstracts for eligibility. Of these, 23 articles were then evaluated for full-text articles. After reading the full text of 23 remaining studies in detail, we eventually identified 16 articles [20–28, 34–39, 42] met the inclusive criteria. Among these, the article by Napier et al. [20] has two RCTs and the article by Peng et al. [34] has multiple comparisons. The PRISMA flow diagram detailing our literature search is illustrated in Fig. 1. All selected studies were in English or Chinese and were published between 2003 and 2018. The key characteristics of RCTs included in the meta-analysis are illustrated in Table 1, while study treatment protocol of RCTs included in the meta-analysis is shown in Table 2.

**Table 1:** Key characteristics of RCTs included in the meta-analysis

Studies	Type	Study period	Sample size		Mean age (mean)		Gender (F/M)		Disease					
									Flexion			Extension		
			Flexion	Extension	Flexion	Extension	Flexion	Extension	OA	RA	PO	OA	RA	PO
Ong 2003 [42]	RCT	2000	20	20	71	74	12/8	13/7	20	0	0	20	0	0
Ma 2008 [27]	RCT	2005–2006	49	46	71	70.6	25/24	22/24	46	3	0	42	4	0
Li 2017 [24]	RCT	2011–2012	54	54	72.6	71.7	43/11	46/8	47	7	0	44	10	0
Liu 2015 [22]	RCT	2013–2014	50	50	73.1	72.4	34/16	32/18	50	0	0	50	0	0
Zeng 2018 [26]	RCT	2016–2017	30	30	68.78	69.68	21/9	20/10	NA	NA	0	NA	NA	0
Panni 2014 [25]	RCT	2012–2013	50	50	69	61	38/12	40/10	50	0	0	50	0	0
Yang 2015 [23]	RCT	2012–2014	23	23	73.6	72.5	11/12	10/13	23	0	0	23	0	0
Antinolfi 2014 [28]	RCT	NA	20	20	73	70.7	13/7	10/10	20	0	0	20	0	0
Napier 2014 I [20]	RCT	2003–2004	86	89	70.4	71	64/22	58/32	78	5	3	78	6	5
Napier 2014 II [20]	RCT	2006	134	140	68.9	70.9	83/51	101/39	131	3	0	137	3	0
Li 2012 [21]	RCT	2008	55	55	71	70	40/15	38/17	47	8	0	46	9	0
Zhao 2014 [36]	RCT	2011–2013	111	102	64.2	63.2	111/0	102/0	111	0	0	102	0	0
Shen 2016 [38]	RCT	2013–2016	90	90	61.9	62.3	54/36	53/37	90	0	0	90	0	0
Hu 2013 [35]	RCT	2010–2012	65	68	67.7	67.8	65/0	68/0	65	0	0	68	0	0
Li 2016 [39]	RCT	2015	50	50	63.1	62.4	16/34	18/32	50	0	0	50	0	0
Guo 2013 [37]	RCT	2012	30	30	72	71	23/7	22/8	30	0	0	30	0	0
Peng 2016 I [34]	RCT	2014–2015	30	30	73.5	73.0	18/12	19/11	30	0	0	30	0	0
Peng 2016 II [34]	RCT	2014–2015	30	30	74.1	73.0	17/13	19/11	30	0	0	30	0	0

*Abbreviation*s: *OA* osteoarthritis, *RA* rheumatoid arthritis, *PO* patellofemoral osteoarthritis, *NA* not available

**Table 2:** Study intervention protocol of RCTs included in the meta-analysis

Studies	Country	Intervention method					Approach	Tourniquet	Drain	TXA	Prophylactic anticoagulation	TT
		Flexion			Extension							
		Knee	Hip	Time	Knee	Hip						
Ong 2003 [42]	the UK	70°	35°	6 h	FE	0°	NA	Yes	Yes	NA	Enoxaparin or aspirin	8 g/dl
Ma 2008 [27]	Australia	70°	70°	24 h	FE	0°	MP	NA	Yes	NA	Enoxaparin 20 mg	NA
Li 2017 [24]	China	< 30°	LE-25	72 h	FE	LE-25	NA	Yes	Yes	NA	LMWH	8 g/dl
Liu 2015 [22]	China	45°	45°	48 h	FE	NA	MP	No	No	NA	Enoxaparin 40 mg	8 g/dl
Zeng 2018 [26]	China	90–60°	NA	24 h	FE	NA	MP	Yes	Yes	Yes	LMWH	7 g/dl
Panni 2014 [25]	Italy	90°	45°	6 h	FE	NA	MP	Yes	Yes	Yes	4000 IU LMWH	8 g/dl
Yang 2015 [23]	China	60°	60°	48 h	FE	NA	MP	No	Yes	Yes	LMWH	9 g/d
Antinolfi 2014 [28]	Italy	90–50°	NA	6 h	FE	NA	MIP	Yes	Yes	NA	4000 IU LMWH	NA
Napier 2014 I [20]	the UK	120°	NA	6 h	FE	NA	MP	Yes	No	No	Aspirin 150 mg	7 g/dl
Napier 2014 II [20]	the UK	120°	NA	6 h	FE	NA	MP	Yes	No	No	Aspirin 150 mg	7 g/dl
Li 2012 [21]	China	30°	30°	72 h	FE	30°	MV	No	No	NA	LMWH	9 g/dl
Zhao 2014 [36]	China	30°	45°	24 h	FE	0°	MP	Yes	Yes	NA	Rivaroxaban(10 mg/d)	9 g/dl
Shen 2016 [38]	China	70°	45°	12 h	FE	0°	NA	NA	Yes	NA	NA	NA
Hu 2013 [35]	China	70°	45°	12 h	FE	0°	NA	Yes	Yes	NA	Rivaroxaban(10 mg/d)	NA
Li 2016 [39]	China	45°	45°	48 h	FE	NA	MP	Yes	Yes	NA	Enoxaparin 0.4 ml	7 g/dl
Guo 2013 [37]	China	70°	30°	6 h	FE	0°	MP	Yes	Yes	NA	Rivaroxaban(10 mg/d)	8 g/dl
Peng 2016 I [34]	China	30°	30°	72 h	FE	30°	MP	Yes	Yes	NA	Rivaroxaban(10 mg/d)	NA
Peng 2016 II [34]	China	60°	30°	72 h	FE	30°	MP	Yes	Yes	NA	Rivaroxaban(10 mg/d)	NA

*Abbreviations*: *LE-25* leg elevated 25 cm, *90–60°* 90° flexion position for the first 12 h and 60° flexion position for the next 12 h, *90–50°* 90° flexion position for the first 3 h and 50° flexion position for the next 3 h, *FE* full extension, *NA* not available, *MP* medial parapatellar approach, *MIP* modified Insallr approach, *MV* mid-vastus approach, *LMWH* low molecular weight heparin, *TXA* tranexamic acid, *TT* transfusion trigger

### Quality assessment and risk of bias

**Fig. 2:**
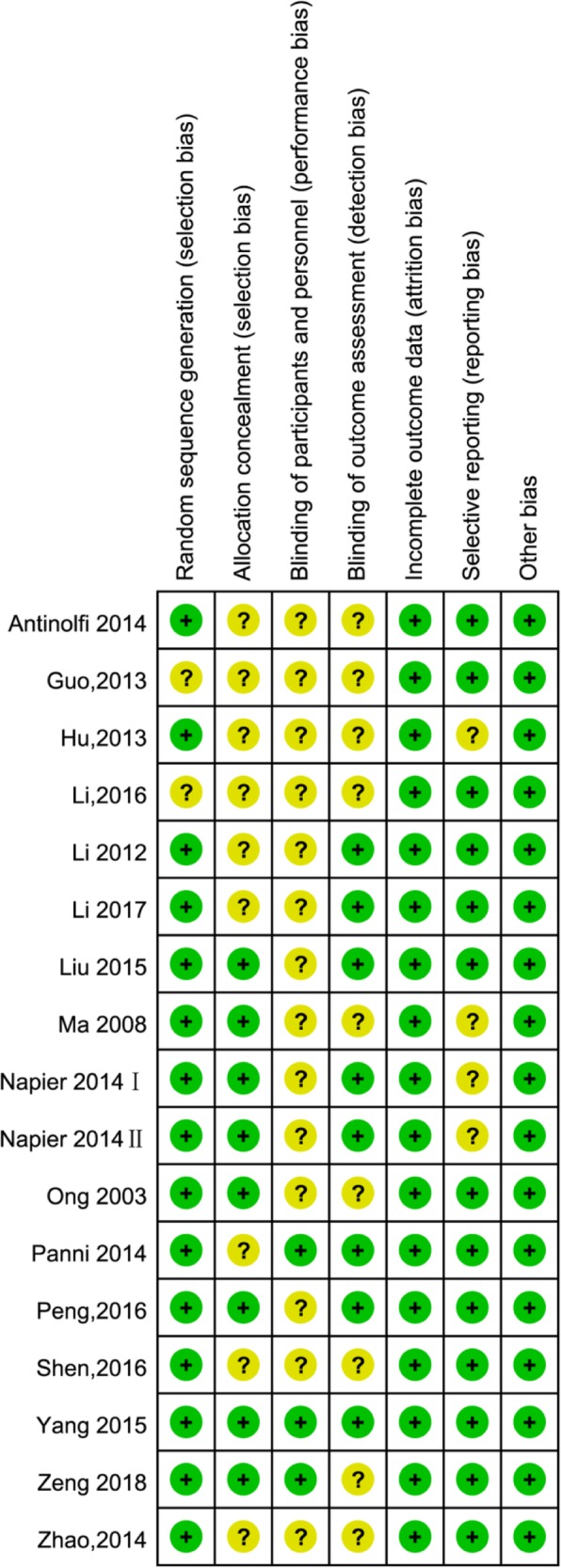
The risk of bias summary of the included studies (+ represents yes; – represents no; ? represents not clear)

**Fig. 3:**
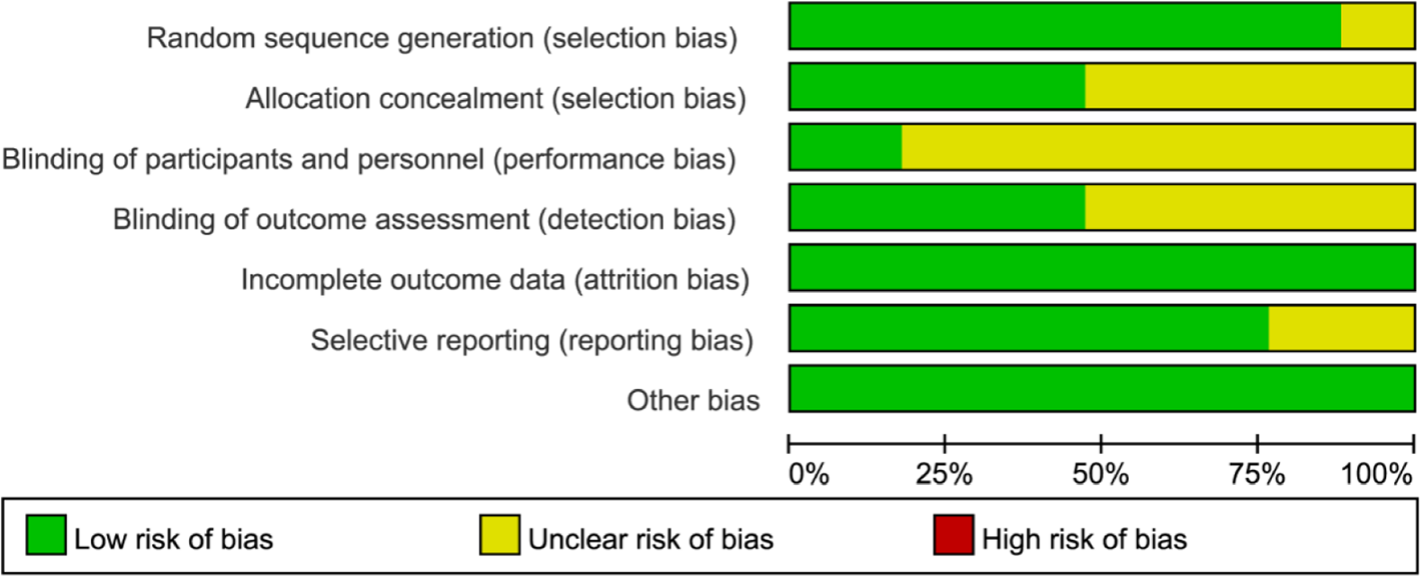
The risk of bias graph of the included studies

The methodological quality of all the included RCTs was evaluated based on the Cochrane Handbook for Systematic Reviews of Interventions. Fifteen RCTs mentioned the adequate randomization technique including random number list [21–25, 28, 34–36, 38], computer-generated block randomization [20, 26], and sealed random number envelope [27, 42]. Allocation concealment was mentioned in 8 trials [20, 22, 23, 26, 27, 34, 42] and unclear in 9 trials [21, 24, 25, 28, 35–39]. Blinding of participants and personnel were mentioned in 3 trials [23, 25, 26] and unclear in 14 trials [20–22, 24, 26–28, 34–39, 42]. Outcome assessors were blinded in 8 trials [20–25, 34], but the blinding in the other 9 trials was unclear [26–28, 35–39, 42]. All included studies furnished complete data and were considered at low risk for attrition bias. The detailed risk of bias of methodological quality in the eligible RCTs is summarized in Figs. 2 and 3.

### Primary outcomes

#### Total blood loss

**Fig. 4:**
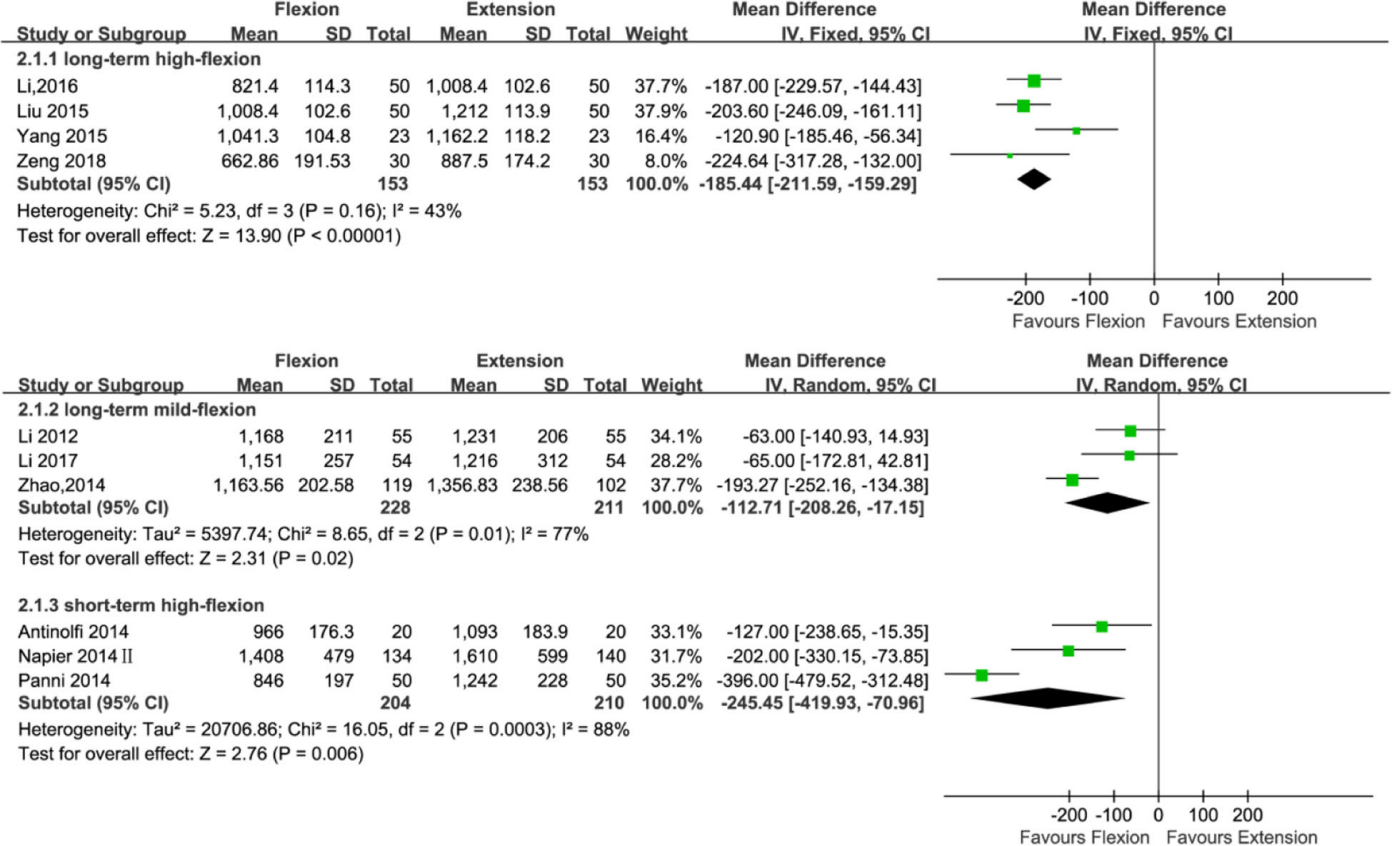
Forest plot for the comparison of total blood loss among the three subgroups

Total blood loss was mentioned in 10 studies (439, 306, and 414 patients in the long-standing mild flexion, long-standing high flexion, and short-standing high flexion groups, respectively). Three studies [21, 24, 36] adopted the postoperative knee management protocols for long-term (≥ 24 h) mild flexion (≤ 30°), four studies [22, 23, 26, 39] adopted the postoperative knee management protocols for long-term (≥ 24 h) high flexion (> 30°), and three studies [20, 25, 28] adopted the postoperative knee management protocols for short-term (< 24 h) high flexion (> 30°). Subgroup analysis showed significant reduction in total blood loss was found using a long-term (≥ 24 h) mild flexion (≤ 30°) protocol (MD = − 112.76; 95% CI, − 208.26 to − 17.15; *P* = 0.02; *I*^2^ = 77%); significant reduction in total blood loss was also found using a long-term (≥ 24 h) high flexion (> 30°) protocol (MD = − 185.44; 95% CI, − 211.59 to − 159.29; *P* < 0.00001; *I*^2^ = 43%) and using a short-term (< 24 h) high flexion (> 30°) protocol (MD = − 245.45; 95% CI, − 419.93 to − 70.96; *P* = 0.006; *I*^2^ = 88%) (Fig. 4 and Table 3).

**Table 3:** Results of subgroup analyses based on diverse postoperative knee flexion protocols

Clinical results	No. of trials	No. of participants			MD/RD	95% CI	*P* value	Heterogeneity *I*^2^	Model
		Flexion	Extension	Total					
Total blood loss									
LH group	4	153	153	306	− 185.44^a^	− 211.59 to − 159.29	*< 0.00001*	43	Fixed
LM group	3	228	211	439	− 112.76^a^	− 208.26 to − 17.15	*0.02*	77*	Random
SH group	3	204	210	414	− 245.45^a^	− 419.93 to − 70.96	*0.006*	88*	Random
Hidden blood loss									
LH group	3	123	123	246	− 95.77^a^	− 138.21 to − 53.32	*< 0.00001*	85*	Random
LM group	3	228	211	439	− 83.70^a^	− 186.28 to 18.88	0.11	98*	Random
SH group	2	80	80	160	− 196.35^a^	− 259.06 to − 133.64	*< 0.00001*	87*	Random
Blood transfusion requirement									
LH group	6	232	229	461	− 0.08^b^	− 0.14 to − 0.03	*0.003*	40	Fixed
LM group	2	70	70	140	7.00^b^	4.77 to 9.23	*0.02*	0	Fixed
SH group	2	84	84	168	0.02^b^	− 0.07 to 0.12	0.62	0	Fixed
ROM (1 week after operation)									
LH group	1	30	30	60	13.00	9.42 to16.58	*< 0.00001*	NA	–
LM group	4	258	241	499	4.75^a^	3.10 to 6.40	*< 0.00001*	43	Fixed
SH group	3	205	208	413	2.63^a^	− 2.80 to 8.06	0.34	93*	Random
DVT									
LH group	6	232	229	461	0.00^b^	− 0.02 to 0.02	1	0	Fixed
LM group	4	258	241	499	0.00^b^	− 0.02 to 0.02	1	0	Fixed
SH group	4	175	179	354	0.00^b^	− 0.03 to 0.03	0.98	0	Fixed
Wound-related infection									
LH group	6	232	229	461	0.01^b^	− 0.03 to 0.05	0.67	0	Fixed
LM group	3	204	187	391	0.00^b^	− 0.02 to 0.02	1	0	Fixed
SH group	4	224	230	454	− 0.00^b^	− 0.03 to 0.02	0.74	0	Fixed

Data in italics indicate a statistically significant *P* value

*Abbreviations*: *LH* long-term (≥ 24 h) high flexion (> 30°), *LM* long-term (≥ 24 h) mild flexion (≤ 30°), *SH* short-term (< 24 h) high flexion (> 30°), *CI* confidence interval, *NA* not applicable

^*^Heterogeneity was statistically significant

^a^Mean difference (MD)

^b^Risk difference (RD)

#### Hidden blood loss

**Fig. 5:**
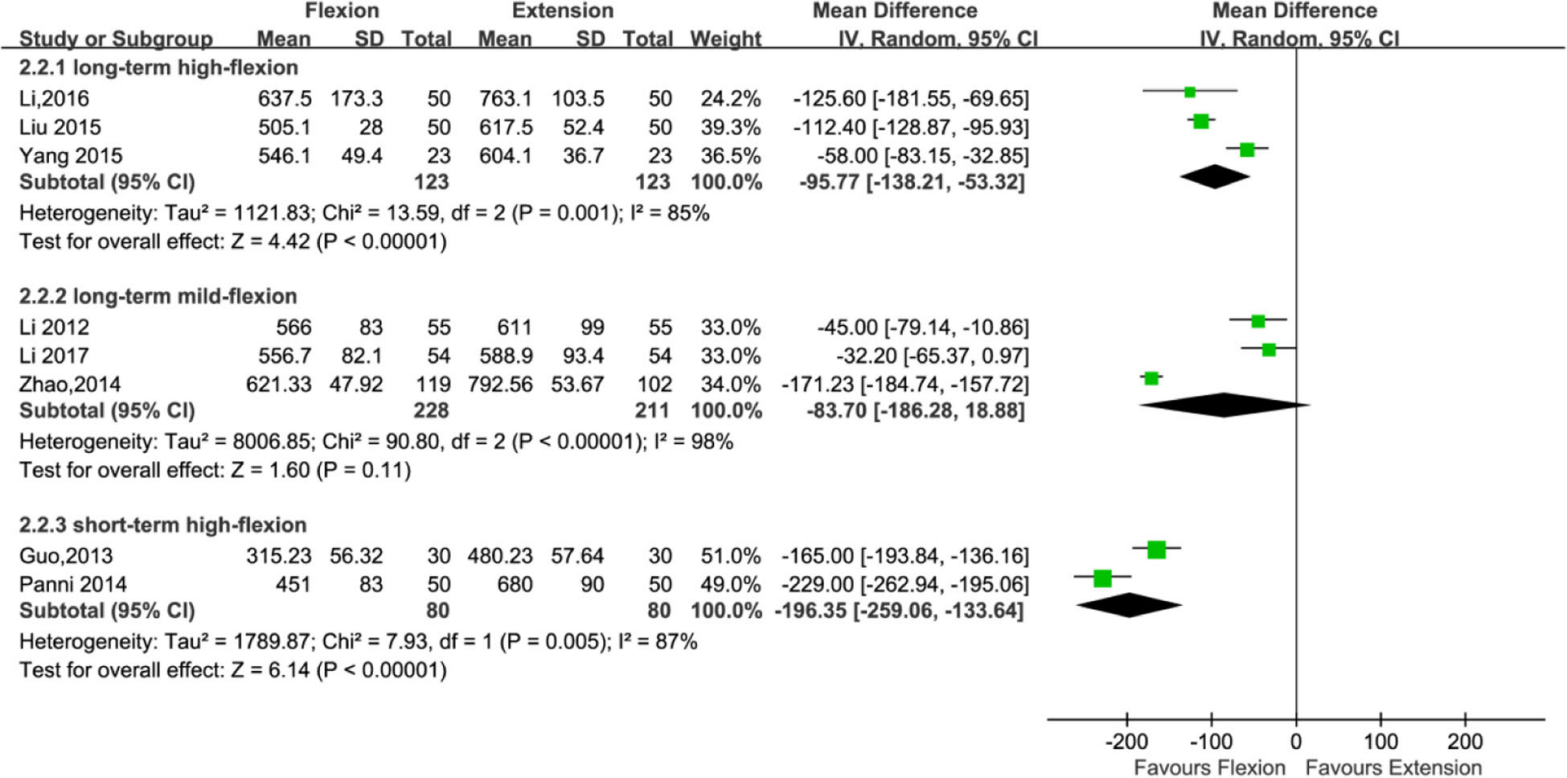
Forest plot for the comparison of hidden blood loss among the three subgroups

Eight studies with a total of 845 patients reported the outcome of hidden blood loss (439, 246, and 160 patients in the long-standing mild flexion, long-standing high flexion, and short-standing high flexion groups, respectively). Three studies adopted [21, 24, 36] the postoperative knee management protocols for long-term (≥ 24 h) mild flexion (≤ 30°), three studies [22, 23, 39] adopted the postoperative knee management protocols for long-term (≥ 24 h) high flexion (>30°), and two studies [25, 37] adopted the postoperative knee management protocols for short-term (< 24 h) high flexion (> 30°). The subgroup analysis revealed that hidden blood loss can significantly reduce using a long-term (≥ 24 h) high flexion (> 30°) protocol (MD = − 95.77; 95% CI, − 138.21 to − 53.32; *P* < 0.00001; *I*^2^ = 85%) and using a short-term (< 24 h) high flexion (> 30°) protocol (MD = − 196.35; 95% CI, − 259.06 to − 133.64; *P* < 0.00001; *I*^2^ = 87%); however, it cannot significantly reduce using a long-term (≥ 24 h) mild flexion (≤ 30°) protocol (MD = − 83.70; 95% CI, − 186.28 to 18.88; *P* = 0.11; *I*^2^ = 98%) (Fig. 5 and Table 3).

#### Blood transfusion requirement

**Fig. 6:**
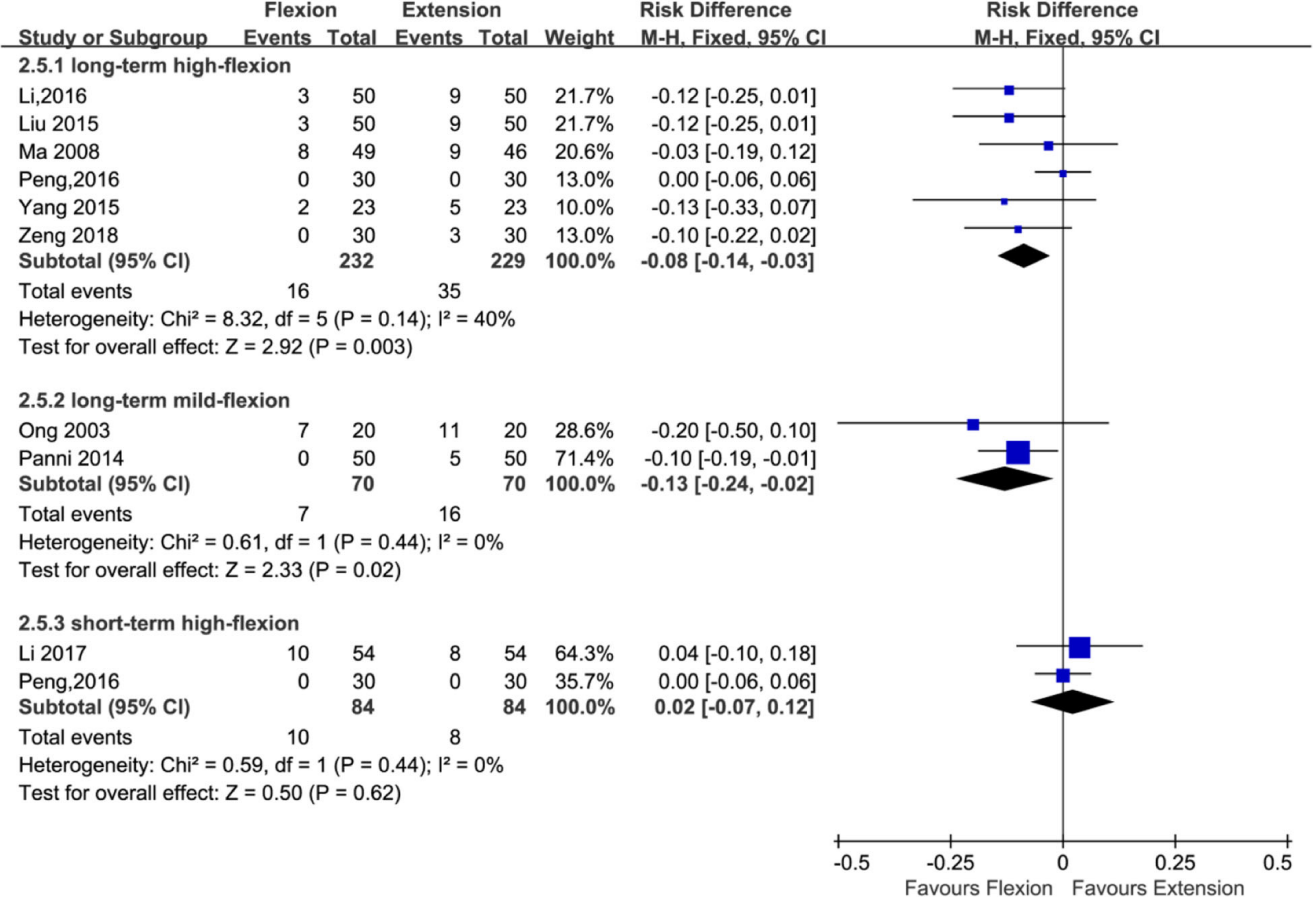
Forest plot for the comparison of blood transfusion requirement among the three subgroups

Ten studies with a total of 769 patients provided the data of blood transfusion requirement (168, 461, and 140 patients in the long-standing mild flexion, long-standing high flexion, and short-standing high flexion groups, respectively). Two studies adopted [24, 34] the postoperative knee management protocols for long-term (≥ 24 h) mild flexion (≤ 30°), six studies [22, 23, 26, 27, 34, 39] adopted the postoperative knee management protocols for long-term (≥ 24 h) high flexion (> 30°), and two studies [25, 42] adopted the postoperative knee management protocols for short-term (< 24 h) high flexion (> 30°). Subgroup analysis that showed no significant reductions in blood transfusion requirement was found when the knee was fixed in high flexion (> 30°) for < 24 h (RD = 0.02; 95% CI, − 0.07 to 0.12; *P* = 0.62; *I*^2^ *=* 0%); however, significant reductions in blood transfusion requirement was found when the knee was fixed in high flexion (> 30°) for ≥ 24 h (RD = − 0.08; 95%CI, − 0.14 to − 0.03; *P* = 0.003; *I*^2^ = 40%) and when the knee was fixed in mild flexion (≤ 30°) for ≥ 24 h (RD = 7.00; 95% CI, 4.77 to 9.23; *P* = 0.02; *I*^2^ = 0%) (Fig. 6 and Table 3).

### Secondary outcomes

#### ROM at 1 week after operation

**Fig. 7:**
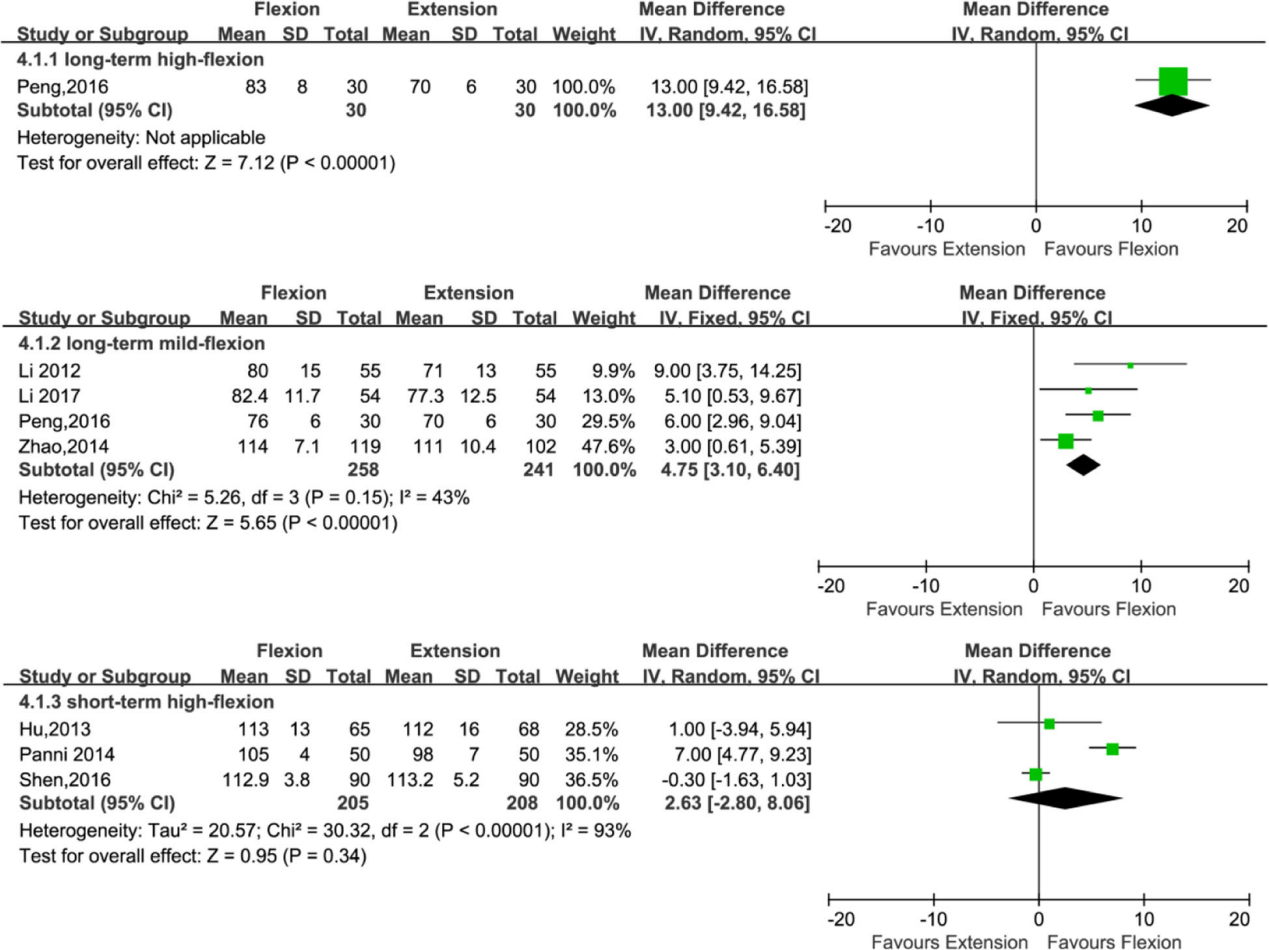
Forest plot for the comparison of ROM at 1 week after operation among the three subgroups

Data from eight studies including 972 patients were available for ROM at 1 week after operation (499, 60, and 413 patients in the long-standing mild flexion, long-standing high flexion, and short-standing high flexion groups, respectively). Four studies [21, 24, 34, 36] adopted the postoperative knee management protocols for long-term (≥ 24 h) mild flexion (≤ 30°); one study adopted the postoperative long-term (≥ 24 h) high flexion (> 30°) protocols; and three studies [25, 35, 38] adopted the postoperative knee management protocols for short-term (< 24 h) high flexion (> 30°). Subgroup analysis that showed no significant improvements in ROM at 1 week after operation was found when the knee was fixed in high flexion (> 30°) for < 24 h (MD = 2.63; 95% CI, − 2.80 to 8.06; *P* = 0.34; *I*^2^ = 93%); however, significant improvements in ROM at 1 week after operation was found when the knee was fixed in high flexion (> 30°) for ≥ 24 h (MD = 13.00; 95%CI, 9.42 to 16.58; *P* < 0.00001; *I*^2^ not applicable) and when the knee was fixed in mild flexion (≤ 30°) for ≥ 24 h (MD = 4.75; 95% CI, 3.10 to 6.40; *P* < 0.00001; *I*^2^ = 43%) (Fig. 7 and Table 3).

#### DVT

**Fig. 8:**
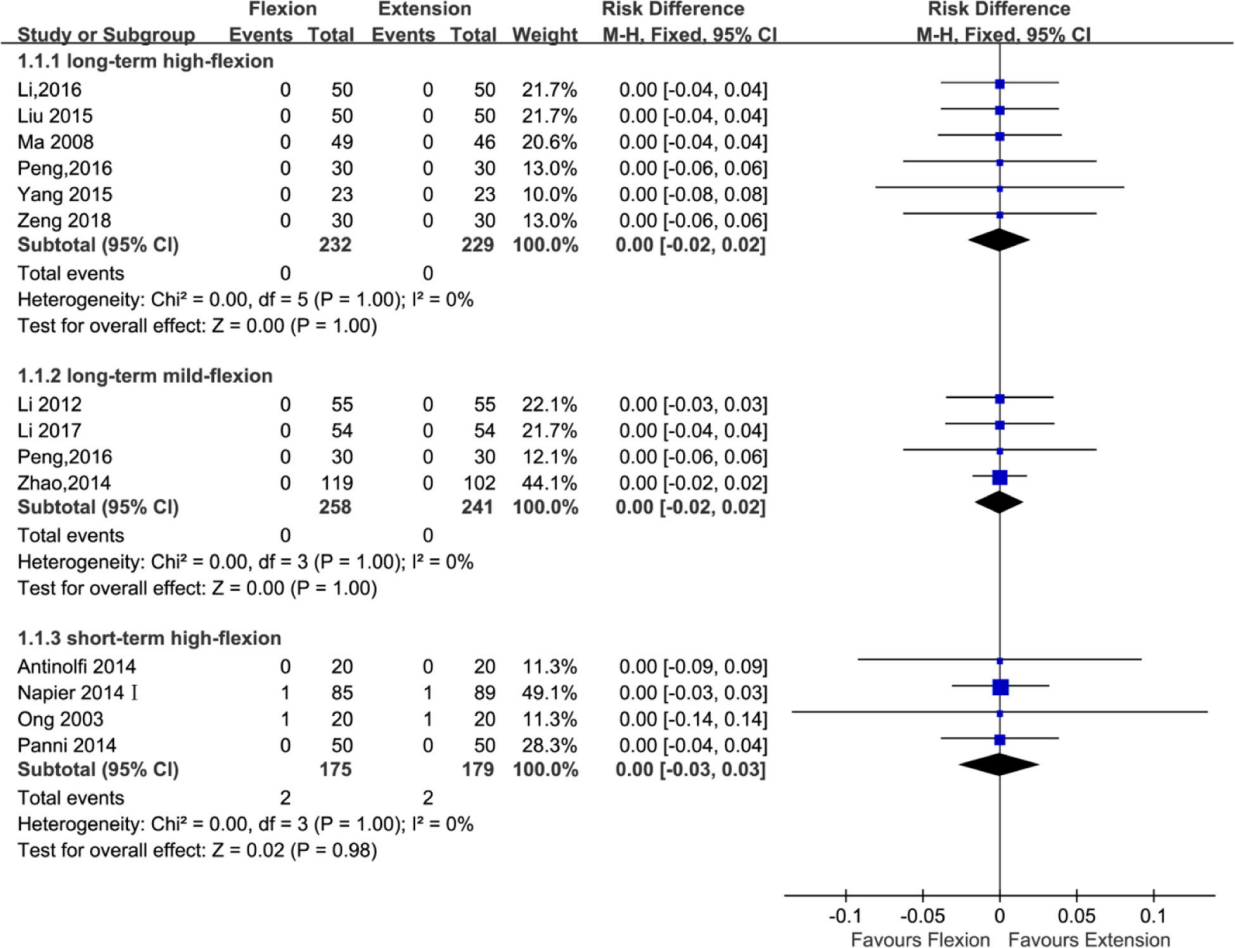
Forest plot for the comparison of DVT among the three subgroups

Fourteen studies with a total of 1314 patients reported the outcome of DVT (499, 461, and 354 patients in the long-standing mild flexion, long-standing high flexion, and short-standing high flexion groups, respectively). Four studies [21, 24, 34, 36] adopted the postoperative knee management protocols for long-term (≥ 24 h) mild flexion (≤ 30°), six studies [22, 23, 26, 27, 34, 39] adopted the postoperative knee management protocols for long-term (≥ 24 h) high flexion (> 30°), and four studies [20, 25, 28, 42] adopted the postoperative knee management protocols for short-term (< 24 h) high flexion (> 30°). The subgroup analysis revealed that DVT cannot significantly reduce using a long-term (≥ 24 h) high flexion (>30°) protocol (RD = 0.00; 95% CI, − 0.02 to 0.02; *P* = 1; *I*^2^ = 0%) and using a short-term (< 24 h) high flexion (> 30°) protocol (RD = 0.00; 95%CI, − 0.03 to 0.03; *P* = 0.98; *I*^2^ = 0%) and that it cannot also significantly reduce using a long-term (≥ 24 h) mild flexion (≤ 30°) protocol (RD = 0.00; 95%CI, − 0.02 to 0.02; *P* = 1; *I*^2^ = 0%) (Fig. 8 and Table 3).

#### Wound-related infection

**Fig. 9:**
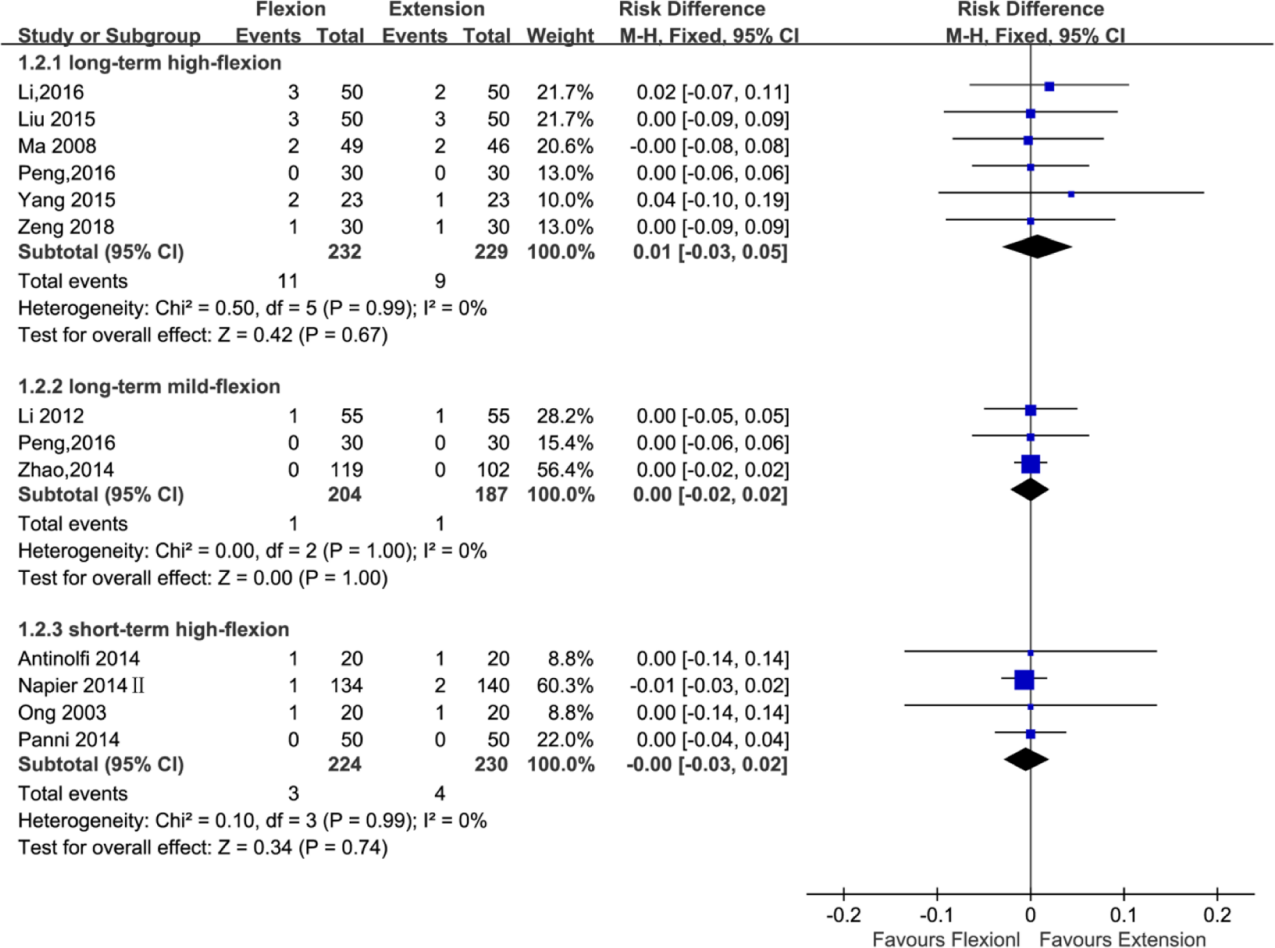
Forest plot for the comparison of wound-related infection among the three subgroups

Thirteen studies with a total of 1306 patients provided the data of wound-related infection (391, 461, and 454 patients in the long-standing mild flexion, long-standing high flexion, and short-standing high flexion groups, respectively). Three studies [21, 34, 36] adopted the postoperative knee management protocols for long-term (≥ 24 h) mild flexion (≤ 30°), four studies [22, 23, 26, 27, 34, 39] adopted the postoperative knee management protocols for long-term (≥ 24 h) high flexion (> 30°), and four studies [20, 25, 28, 42] adopted the postoperative knee management protocols for short-term (< 24 h) high flexion (> 30°). The subgroup analysis revealed that wound-related infection cannot significantly reduce using a long-term (≥ 24 h) high flexion (> 30°) protocol (RD = 0.01; 95%CI, − 0.03 to 0.05; *P* = 0.67; *I*^2^ = 0%) and using a short-term (< 24 h) high flexion (> 30°) protocol (RD = − 0.00; 95%CI, − 0.03 to 0.02; *P* = 0.74; *I*^2^ = 0%) and that it can also not dramatically reduce using a long-term (≥ 24 h) mild flexion (≤ 30°) protocol (RD = 0.00; 95%CI, − 0.02 to 0.02; *P* = 1; *I*^2^ = 0%) (Fig. 9 and Table 3).

### Publication bias

**Fig. 10:**
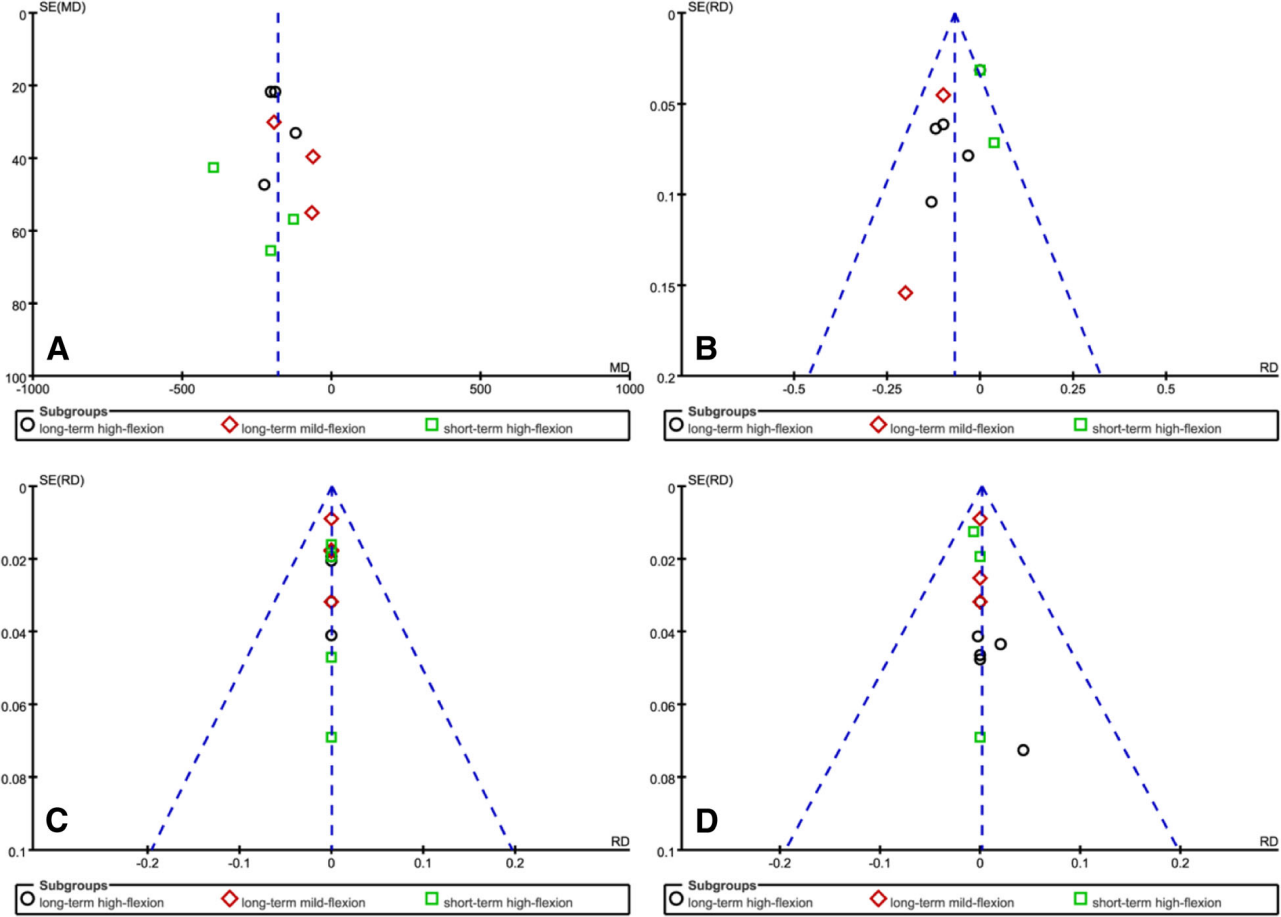
Funnel plot of the current meta-analysis. Total blood loss (**a**). Blood transfusion requirement (**b**). DVT (**c**). Wound-related infection (**d**)

Four funnel plots based on total blood loss, blood transfusion requirement, DVT, and wound-related infection was used to assess publication bias and demonstrated minimal asymmetry and a few outliers, indicating minimal evidence of publication bias (Fig. 10)

## Discussion

This meta-analysis compared the effect of different postoperative knee flexion protocols on patient’s outcomes after primary TKA. To our knowledge, this is the first meta-analysis of RCTs that evaluated the effectiveness and safety based on both the degree of knee flexion and duration of knee flexion following primary TKA. The most important finding of the present study is that long-term (≥ 24 h) high flexion (> 30°) protocol could not only significantly reduce total blood loss, hidden blood loss, and transfusion requirements but could also effectively improve ROM at 1 week after operation; short-term (< 24 h) high flexion (> 30°) protocol could significantly reduce total blood loss and hidden blood loss; long-term (≥ 24 h) mild flexion (≤ 30°) significantly reduce total blood loss and transfusion requirements and improve ROM at 1 week after operation. There was no significant difference with respect to the incidence of wound-related infection and DVT between the three flexion groups during the follow-up period.

Blood loss in TKA is a significant problem for the orthopedic surgeon who can use a variety of perioperative methods to minimize it and possibly reduce any related side effects. Two previous meta-analyses have shown that compared to knee full extension, postoperative flexion position is a simple and cost-effective approach to reduce blood loss and transfusion requirements and increase ROM in the early postoperative period after primary TKA [7, 30]. Theoretically, the knee position was closely related to the tension of popliteal vessels. The postoperative knee flexion, to some extent, lowered the tension of popliteal vessels, which can increase venous return [24]. This process takes turns to reduce total blood loss and hidden blood loss including blood extravasation into the tissues and residual blood in the joint, which can distinctly lower intraarticular pressure and capsular tension, ultimately accelerating range of motion restoration [21, 23, 25, 33]. However, there remains no consensus upon whether it is specific knee position and duration of knee flexion which conduces most to the benefits seen with postoperative knee flexion protocol. Long-term knee flexion may be beneficial to reduce blood loss [25]. Faldini et al. [31] performed a systematic review of seven studies to assess the effect of postoperative limb position on outcomes after TKA and found that a 48–72 h postoperative knee flexion appears to be beneficial to reducing blood loss and increasing ROM following TKA; short-term flexion protocol failed to alter these parameters. Mild flexion may be less effective for blood loss reduction because it leads to lower compression on the blood vessel with lower effect on peripheral blood circulation [25]. However, Wu et al. [32] performed a meta-analysis of nine RCTs to compare the effectiveness of different limb positions in primary TKA and found that knee mild flexion (< 60°) protocol is significantly beneficial with hidden blood loss after TKA compared with knee high flexion (≥ 60°); knee high flexion (≥ 60°) protocol is superior to mild flexion (< 60°) protocol in reducing blood transfusion requirements and improving ROM following TKA. De Fine et al. [33] conducted a randomized controlled trial of 62 patients to understand the optimal degree of flexion required to improve functional outcomes and found that no significant differences were found between the high flexion (70°) and mild flexion (30°) group in terms of blood loss, transfusion requirements, and ROM after TKA. In the present meta-analysis, although the differences in total blood loss between three flexion protocols were unclear, subgroup analysis suggested that long-term (≥ 24 h) high flexion (> 30°) protocol could provide extra benefits compared with short-term (< 24 h) high flexion (> 30°) protocol in regard to ROM at 1 week after operation and compared with long-term (≥ 24 h) mild flexion (≤ 30°) protocol regarding hidden blood loss and blood transfusion requirements. Nevertheless, there was significant heterogeneity in terms of total blood loss and hidden blood loss in the three flexion subgroups. Thus, we used a random effects model to analyze these statistical data. But, we speculate that the observed heterogeneity was mainly caused by the clinical differences, such as differences in the operating skill of different surgeons and differences in the application of the tourniquet, drainage, and tranexamic acid.

Wound-related infection is one of complication following TKA. Johnson has reported that keeping the knee in flexion position following TKA may maximize wound complications by reducing oxygen tension on the skin edges [43]. However, the decreased oxygen tension at the skin edges secondary to postoperative mild knee flexion should be compromised by the increased local perfusion and oxygen tension favored by the reduction in knee swelling [21, 25]. Subgroup analysis in this meta-analysis showed that the significant difference in the rate of wound-related infection was not found between three flexion protocols. A greater degree of knee flexion may curve popliteal veins and hinder venous return [44]. Obstructed venous return resulted from knee flexion should increase the risk of DVT [24]. But, subgroup analysis of this meta-analysis found that the no significant difference in the rate of DVT was not found between different flexion protocols.

Our meta-analysis has several advantages compared with the meta-analysis previously published by Faldini et al. [31], Fu et al. [7], Jiang et al. [30], and Wu et al. [32]. First, our study included one recent high-quality RCT by Zeng et al. [26] and six RCTs [34–39] written in Chinese excluded by previous reviews, which would have reduced statistical bias and publication bias. Second, our study also excluded one [29] quasi-randomized trials and two earlier trials lacking of specific data in knee flexion angle [45, 46], and subgroup analysis based on both the degree of knee flexion and duration of knee flexion was conducted, bringing about more precise conclusions. Third, we used a funnel plot to assess publication bias when the number of trials reporting the primary outcomes was ten or more, and these results indicated that publication bias was well controlled. Therefore, these factors strengthen the availability of our findings.

However, our meta-analysis also has some limitations. First, the majority of RCTs we included were characterized by their small sample size and the number of RCTs included in each knee flexion subgroups remains too small, which might increase the likelihood of misestimation magnitude of the intervening effect. Second, heterogeneity among the included studies was unavoidable because of transfusion trigger differences, differences in the operating skill of different surgeons, and differences in the application of the tourniquet, drainage, anticoagulant, and tranexamic acid. Third, the follow-up duration of DVT in the included studies was different, which may have influenced the rate of postoperative DVT. Fourth, we failed to obtain partial original data from some included study authors by e-mail, such as drainage blood loss, length of stay, hospitalization costs, which hinder us from fully analyzing these statistical data between different flexion protocols.

## Conclusion

This meta-analysis showed that the long-term (≥ 24 h) high flexion (> 30°) protocol could be optimal limb management to reduce blood loss and blood transfusion requirements and facilitate early postoperative rehabilitation exercises in patients after primary TKA without increasing in complication rate. However, considering the defect of the study design, several biases, such as selection bias, performance bias, and detection bias, may lower the hierarchy of evidence quality. So, more well-designed and large-scale RCTs with long-term follow-up that identifies the efficacy and safety of different postoperative knee flexion protocols in patient’s outcomes will be needed in the future.

## Abbreviations

CI: Confidence interval
DVT: Deep vein thrombosis
MD: Mean difference
PRISMA: Preferred Reporting Items for Systematic Reviews and Meta-Analyses
RCTs: Randomized controlled trials
RD: Risk difference
ROM: Range of motion
TKA: Total knee arthroplasty
